# Stigma among healthcare workers towards hepatitis B infection in Bangalore, India: a qualitative study

**DOI:** 10.1186/s12913-019-4606-z

**Published:** 2019-10-22

**Authors:** F. C. van der Scheun, M. C. M. Nagelkerke, A. Kilaru, V. Shridhar, R. Prasad, T. S. van der Werf

**Affiliations:** 1 University of Groningen, Faculty of Medical Sciences, Antonius Deusinglaan 1, 9713 AV Groningen, the Netherlands; 2 PCMH Restore Health Center, Bangalore, India; 3 Academy of Family Physicians of India (AFPI), Karnataka, India

**Keywords:** Hepatitis B, Stigma, Doctors, Nurses, India, Bangalore, Qualitative, HIV, Awareness

## Abstract

**Background:**

With about 50 million people infected with hepatitis B (HBV) in India the burden of disease is high. Stigma has been identified to have a major negative impact on screening, diagnosis and treatment of hepatitis B patients. The aim of this study was to assess the stigma in nurses and physicians in Bangalore, India; studies on stigma in HBV have only been published outside of India.

**Methods:**

Semi-structured in-depth-interviews were conducted in the period of March 20th and April 16th 2018 to study stigma and other problems in the care of hepatitis B patients. Stigma was pragmatically defined as a mark of disgrace associated with having a hepatitis B infection. Thirty physicians and nurses in different clinics and hospitals across the city of Bangalore were selected by purposeful sampling and snowball effect until theoretical saturation was reached.

**Results:**

The following themes were identified during the interviews: feelings when treating a patient; pregnancy and marriage; confidentiality; morality; improvement in care and the difference with HIV. The most stigma was discovered in the theme morality. The majority of our participants mentioned lack of awareness as biggest obstacle in health care of hepatitis B patients.

**Conclusions:**

This is the first qualitative study in India exploring hepatitis B stigma among health care workers. Stigma was found in certain themes, such as morality. Though, no unwillingness to treat was found. There was a general lack of awareness amongst patients according to our participants and could jeopardize proper treatment. These results will further help in developing strategies to tackle hepatitis B in India.

## Background

Hepatitis B virus (HBV) remains a significant global health problem with 292 million people infected worldwide [1]. Even though effective vaccination is available, the decline in prevalence globally is only happening in a gradual manner and the overall burden remains extremely high. In India, studies report an overall prevalence of 1.3–7% with an average of 4%, making the disease burden 50 million [2–5].

Hepatitis B is a viral infection caused by the hepatitis B Virus, a double-stranded DNA virus from the Orthohepadnavirus family. The disease can be transmitted vertically (i.e., from mother to child) and horizontally (i.e., by transmission through blood-blood contact, or by mucosal – usually, sexual intercourse). Most infected persons recover, but 5–10% will become chronically infected. Depending on the persistence of HBV surface antigen (HBsAg) in the serum, the infection varies from acute to chronic [6]. The age of acquiring a hepatitis B infection is a major determent of chronicity. Infants with an infection acquired by vertical transmission have a 90% risk of chronic HBV, compared to 5–10% of adults by horizontal transmission. In India, vertical transmission in childhood seems to play a predominant role, although data is limited [2–4, 6]. Most of the chronically infected develop a mild liver disease with little to no long term mortality and morbidity; others will have cirrhosis with chronic hepatic failure. This can result in death [7, 8]. Patients with chronic HBV can effectively be managed with interferon based and nucleoside analogues. Both reduce virus replication, liver inflammation, and the risk of liver disease [9]. However, these treatment options are expensive and remain unaffordable for many in India. In 2018, an initiative of the government of India was launched, aimed to expand access to diagnostics and medications for the HBV infected [10].

With treatment the viral load will decrease gradually. However, the treatment does not cure the infection and is primarily life-long for most people to whom it is prescribed [11]. Early screening is important, notably to prevent transmission. For adequate follow up, disease knowledge is essential. Proved barriers to management of this chronic infection include poverty, lack of awareness and stigma [12].

Globally, stigma has been identified as a significant barrier for screening, diagnosis and treatment of hepatitis B [12–14]. Stigmatized individuals with this infection can be denied health care as well as education, employment, and interaction with society [15] and therefore also severely harming the mental health and quality of life [16]. Negative attitudes can be expressed by any social relationship. In India, stigma has been reported as an important barrier to accessing care for multiple health conditions, including HIV, tuberculosis, certain types of cancer and hepatitis [17–19]. Among the most important sources of information and care in India are healthcare workers, who have also been reported to be major sources of stigma in HIV, a chronic infection with similar routes of transmission to hepatitis B [20, 21]. Negative attitudes of health care workers have rarely been studied in the case of hepatitis B.

With a population of 1.3 billion inhabitants, India is one of the most HBV-burdened areas around the world [22]. However, specific data on stigma related hepatitis B infection in India is disproportionately low compared to the burden and global significance of the problem. Understanding the impact of stigma in the prevalence of hepatitis B could improve the strategies to tackle the high burden. In this study, we explored stigma among hepatitis B in health workers in the city of Bangalore India by using qualitative methods. We hypothesized that healthcare workers in India express at least some stigma towards hepatitis B-infected individuals.

## Methods

### Study design

This descriptive qualitative study was conducted in the period of March 20th and April 16th 2018. Data collection consisted of conducting semi-structured in-depth-interviews. Through the interviews, data was collected on protocols, work environment, opinions and beliefs about hepatitis B. Stigma was pragmatically defined as a mark of disgrace associated with having hepatitis B infection; as we studied stigma among health care workers, we studied inflicted, not perceived stigma. Although stigma has been studied using quantitative scales we explored stigma in a qualitative way in this exploratory study [14]. Stigma in this context was the accumulation of non-factual ideas and behaviours including shame and blame associated with people living with HBV.

### Study participants

We included nurses and physicians (HCWs) working in Bangalore India. We aimed at enrolling participants highly likely to encounter patients with hepatitis B. Therefore, among physicians we included participants from in the following fields: gastroenterology, general medicine, obstetrics and gynaecology, anaesthesiology and surgery. Among nurses, considering the fact that in India nurses often rotate between departments, no specific inclusion or exclusion criteria were used. We excluded those unwilling to participate, not being able to hear, HCWs who might be biased due to close working with our research team or not being able to speak English when we did not have our translator to help us. In addition, we only used up to 3 participants from one hospital or clinic and selected different types of care to create diversity in our study group. No financial or other incentive or reimbursement was given in any form to participate. Participants were approached face-to-face and in some cases via phone call.

### Data collection

We used purposeful sampling and the snowball effect in our study. Participants were first found on our preselected criteria and social networks to find a wide range of staff in different fields. No relationship was established prior to the study. Subsequently other participants were found through the referral system, to find less easily accessible healthcare workers. We approached hospitals that serve patients from different economic background, so as to include both private clinics and government hospitals. In addition, we included different care systems - primary, secondary and tertiary care facilities in the public and private sector. In hospitals where we suspected that staff members were not English-speaking, we had the help of a translator. The informed consent was translated in common local languages in this area, Kannada, Telugu and Malayalam if necessary for an interview. In the absence of previous studies in this field, we were unable to predetermine a sample size beforehand that would suffice to achieve thematic saturation – as a result, our approach was an exploratory study. The size was determined by our resources, time and theoretical saturation. After piloting, this resulted in a total of 30 face-to-face interviews with physicians and nurses. Each interview lasted between 15 and 35 min and was held in a private room of the hospital with one facilitator present. The interviews continued until theoretical saturation was reached and no new information was revealed. No repeat interviews were conducted. No contact was kept with participants after the interview and no files were returned. We interviewed 16 physicians and 14 nurses. Details of the participants are shown in Table 1.

**Table 1:** Characteristics of study participants

Characteristics		In-depth interviews (*n* = 30)
Gender	Female	19
	Male	11
Type of hospital	Government	14
	Private	16
Type of care	Primary	11
	Secondary	3
	Tertiary	16
Nurses	Staff	13
	Superintendent	1
Doctors	Gastroenterology	1
	Anaesthesiology	1
	Surgery	4
	Obstetrics & Gynaecology	5
	General	4

We included 14 different locations in Bangalore: Ashwini Clinic, Primary Medicare Center, Aster Clinic, Ramesh Clinic, RxDx Healthcare Whitefield, Manipal Hospital, People Tree Hospital, Divakars Speciality Hospital, Bowring and Lady Curzon Hospital, Jayanagar General Hospital, Ejipura PHC, Sita Bhateja Speciality Hospital, Victoria Hospital and Apollo Hospitals. The study population reflected a variety of participants in the different healthcare settings in India.

Two female student researchers (MN, FS) conducted all interviews. A short training was given by the supervisors to explain the rationale of this study related to beliefs and attitudes among HCW related to people living with HBV. The individual semi-structured interviews began by an informal talk on the subject and reasons for the research and any questions asked by the participant. A translator was present on four occasions. In case the participant was illiterate or not able to read and understand the informed consent form, a translator discussed the consent form in the participant’s local language. Sufficient time was given to consider participation, after which the participant was offered to sign the consent. Written informed consent was obtained from every participant. All interviews were confidential and completely voluntary; 13 (29%) subjects screened refused participation. Reasons were not sought. Dependent on the participants, information was read by the individual independently or thoroughly discussed together with the researcher. To maintain confidentiality, no identifiers (like name and age) of any of the participants were recorded. Only location, date, occupation and gender were collected with the audio recording. No field notes were made during data collection. Although age would be interesting for our research, the ethical committee did not allow collecting this identifier; code names were used for every participant. All audio was transcribed with translations if necessary. Transcriptions, audio and other relevant files were only accessible to two researchers (FS, MN) and their supervisors. We used a period of two weeks to pilot the interview guide that was adapted for further use. The natural flow of the conversation led to different topics in each interview. However, the interview guide ensured that certain important aspects were addressed during each interview. The interview guide we used has been added as electronic attachment to this paper (See Additional file 1). Open ended and deepened questions were asked, such as: ‘Can you give a general overview how your clinic/hospital usually treats hepatitis B patients?’, ‘Imagine you have a hepatitis B infected patient, and the partner of this patient doesn’t know. How would you handle this?’ and ‘How do you personally feel about treating a patient who has hepatitis B?’. Follow up questions were used, for instance ‘Tell me more about..’ and ‘Could you tell me why you feel this way..’.

### Data analysis

All data was collected by audio as mentioned above; afterwards these were transcribed. All transcripts were uploaded in ATLAS.ti version 8. This is a qualitative research tool for the analysis of large textual data. All 30 conducted interviews were read through multiple times. Relevant quotations were found and broken down into codes by the two main investigators. Subsequently a coding tree was made and main themes were found.

### Ethical approval

For this research project, a waiver from the Ethical Committee of the UMC Groningen was obtained. Additionally, Ethical approval was also obtained by the Institutional Review Board of Swami Vivekananda Youth Movement, Mysore, India.

## Results

Content analysis of the in depth interviews revealed the following main themes from the coding tree: feelings when treating a patient; pregnancy and marriage; confidentiality; morality; improvement in care and the difference with HIV. The selected themes were identified to understand stigma in health care workers.

### Feelings while treating a patient

Firstly, when asked about feelings while treating, all answered never to refuse a patient with hepatitis B. Fourteen stated not to be scared at all: ‘Just like any patient’ (8.doctor), ‘No difference’ (13.doctor) and ‘All patients are equal’ (29.nurse). Secondly, although 3 nurses felt scared while treating a hepatitis B patient, they did not feel this influenced their work: ‘Scary. But, only scary. Otherwise I don’t have any problem with it. I’m, personally, I love to treat them, because they are also human beings’ (21.nurse). Others only felt they behaved somewhat more cautious while treating a patient, one stated: ‘While drawing any kind of blood, any kind of, you know blood transfer, or transfusion, or even drawing blood for blood samples, she will take extra precautions she will look at her safety’ (31.nurse).

### Morality

Out of the 22 participants that understood the question, 10 participants believed that hepatitis B is transmitted due to “immoral behaviour”. Participants defined immoral behaviour mostly as having multiple sexual partners, using drugs or working in the sex industry. 15 out of 25 individuals saw a hepatitis B infection as the patient’s own fault in certain cases, while the others never saw it that way. Two thought a hepatitis B patient “got what he deserved”. Most participants disagreed. One stated the following: ‘No absolutely not (laughs), not like that at all, it is an infection; it needs to be treated correctly’ (3.doctor).

### Marriage and pregnancy

Most participants had a positive view regarding people with hepatitis B marrying or having children. Of 27 study participants, 24 were positive about discordant marriages. Twice the vaccination was mentioned: ‘Yes you can, but you have to take precautions first. Treatment, get treatment first, and precautions with respect to sexual behaviour’ (8.doctor) and ‘If we are comfortable with vaccinations and all, then we can get married’ (17.doctor). However, for 11 of them this didn’t mean that they could imagine marrying an infected person themselves. Among reasons given were “higher mortality” (1.doctor) or ‘All the time we cannot be that much cautious’ (13.doctor). Some participants had a negative attitude about infected women getting pregnant. However most (92%) were positive: ‘Not a problem’ (7.doctor). Half of these mentioned vaccination in regard to this: ‘Normally no, because it will spread to the kids also. But nowadays they have a vaccine and all’ (22.nurse) and (28.nurse) said: ‘So the woman can get pregnant, but she should take treatment, and the baby should get vaccine’.

### Confidentiality

Most participants stated to make sure not to display a patients’ hepatitis B status in a public place: ‘If we put on the door, then the patient also feeling bad, ma’am’ (20.nurse). Another commented: ‘We have to keep confidentiality, and we can’t put any board, or, separate board, or something, we can’t tell’ (21.nurse). However, when it came to partner disclosure, two thirds of the participants reported that they would inform the partner against the patient’s will. One participant commented the following: ‘If she is ok, we will tell. If she is not ok, also we have a duty to inform the guardians and the people surrounding them’ (10.Doctor). A few more HCWs agreed to tell the surroundings of the patient: ‘Not only partner, everyone in the family should know’ (13.doctor). Explanations included the following: “avoid lots of problems in the future” (16.doctor) and “so that they can all take the precautions” (30.nurse).

### Difference with HIV

Six participants brought up themselves that, even though comparable in transmission routes, hepatitis B is surrounded by much less stigma than HIV: ‘With hep B, I must say, what HIV has created, the fear, it’s not there in hep B’ (8.doctor). Three participants informed us that, while for hepatitis people mostly don’t have a problem informing their partner, for HIV they certainly do. One stating: ‘If someone was found to be HIV positive, literally, we had situation where the whole village leadership would turn up in the hospital and not allowing the person back into the village. That never happens with hep’ (6.doctor).

### Improvements in care

During the conversations about general problems in the care for hepatitis B patients, we asked participants for barriers in care of hepatitis B, “unawareness” was often brought up. Statements included: ‘I don’t think people are aware of the consequences of having hep B, other than medical professionals’ (5.doctor) and **‘**I find two extreme reactions, one totally unaware, not wanting to get vaccinated (..), they hear it can impact the baby (..). And the other one is thinking it is a very bad disease**’** (3.doctor). Some doctors among the study participants felt that this would lead to increased transmission of the disease: ‘We need to make people aware of how it spreads. That is where we can contain it. The people do not know here’ (16.doctor). And - because people do not realize the severity - to loss in follow up. A few HCWs specifically mentioned unawareness related to illiterate patients: ‘It is an illiterate problem, and the drug addict, and unaware of knowledge about medical things’ (25.nurse) and ‘Many are illiterate here, so they don’t know, they need to be educated about it’ (15.doctor). It was also mentioned that in government hospitals, there is often not sufficient time to explain the disease. “Education” was the most prominent improvement suggested by our participants.

## Discussion

This is one of the first qualitative studies from India to assess stigma among HCWs towards persons infected with hepatitis B. Experiences and opinions of our Indian HCWs showed some stigma present mainly in the theme morality. Furthermore, we found relatively low stigma in all other areas. Around half of our participants had a moral judgement about people living with hepatitis B, or even thought this was their own fault. We did not find any study in India regarding this subject. However, our findings are in line with other studies published on perceived stigma by people living with hepatitis B in China and Iran [15, 23]. The negative judgements and opinion by our participants underline the existence of negative attitudes towards people living with hepatitis B. These attitudes proved to consequently negatively influence family, employment, finances and social interactions of persons infected with hepatitis B in China and Iran [15, 23]. Our study does not represent the general population of India, since we only included HCWs. Our participants have knowledge of the infection, which might cause less stigma, as shown in a similar study done in Pakistan [24]. Additionally, our participants are also among the populations that are required to get the triple dose vaccine. Therefore it is possible that there might be more stigma towards people with hepatitis B in real life among the general population.

Even though some stigma can be found in moral judgments of HCWs, there was no reported resistance to treat any patient with hepatitis B. Four participants reported being scared of treating hepatitis B infected patients. Once again, no relevant studies can be found regarding hepatitis B attitudes in India to compare our data. Willingness to treat hepatitis B patients by nurses reported 73.0% in Vietnam and 72% in Japan [25, 26]. First, the study populations were different from ours. In both Japan and Vietnam only nurses were questioned - we also included physicians. Increased knowledge among physicians might explain why we found less stigma and higher willingness to treat [27]. Second, our study used a different methodology. We conducted face-to-face interviews with participants, while the other studies used self-administered questionnaires. Hence, it is possible that our participants were hesitant to admit unwillingness to treat in a face to face interview.

Our study highlights the lack of awareness amongst patients about their own disease. Many participants felt this unawareness was jeopardizing proper treatment, since it caused poor follow up and ongoing transmission. Improved public awareness on transmission routes, screening, and follow-up is clearly needed, especially for pregnant women.

### Limitations

Our study had few limitations - one being a selection bias for participants with an adequate level of English, especially on days that we could not make use of translation services. Furthermore, if during the analysis we felt the interviewee did not understand our question - because of difficulties in handling or understanding English language used - we did not include the answer in our results. In addition, in our study we made use of in depth interviews. People possibly felt hesitation to admit fear or stigma, or would otherwise give socially desirable answers, even though questions were asked to explore in depth people’s feelings. This could explain the differences in stigma: since the previous studies used self-administered questionnaires, participants might have been more daring in giving less socially desirable answers and be more honest.

### Recommendations for further research

Our research highlights the themes around which stigma is notable, for example that of morality, and thus suggests potential aspects to focus on in stigma-reducing health communication and policy. We suggest further research to focus on ways of reducing stigma in these areas. Our participants stated that lack of awareness jeopardizes proper treatment. Further research should therefore also focus on the most effective approaches and tools to increase awareness amongst patients.

Since our study was of a qualitative and exploratory character, we also suggest a quantitative study to confirm our results. A quantitative study could also look at variables influencing stigma such as age, gender, background, social class, religion and geographical region. In addition, this study can make use of a bigger sample size, consequently validated significant differences can be found.

Furthermore, studying stigma from the perspective of hepatitis B positive people would be especially insightful and beneficial to understand more fully its nature and consequences.

## Conclusions

Our study on stigma towards hepatitis B among HCWs is the first in India. In certain areas this stigma is prevalent, for example in morality. This does not seem to influence the willingness to treat a patient with hepatitis B, though it could have impact on the way they treat them. Our results encourage future researches to look at other problems for improving hepatitis B care, such as awareness. More research is needed to focus on effective approaches and tools to reduce to high burden of hepatitis B in India.

## Supplementary information

**Additional file 1.** Interview Guide. List of questions used to guide interviews with participants regarding beliefs and attitudes related to people living with HBV.
