# Supplementary material for: Stigma among healthcare workers towards hepatitis B infection in Bangalore, India: a qualitative study
Source: BMC Health Serv Res. 2019 Oct 22;19:736. doi: 10.1186/s12913-019-4606-z (PMC6805630; doi:10.1186/s12913-019-4606-z)
Supplement: Supplementary file 1 — Additional file 1. Interview Guide. List of questions used to guide interviews with participants regarding beliefs and attitudes related to people living with HBV. [file 12913_2019_4606_MOESM1_ESM.docx]

**Interview Guide**

Time:

Place:

Date:

Interview conducted by:

**Demographics**

1. Gender?

2. What is your occupation in this hospital?

3. What is the highest level of education you received?

4. Do you consider yourself religious?

5. Which religion do you adhere to?

**General questions**

1. Do you personally know anyone with hepatitis B?

2. In what ways is hepatitis B transmitted?

3. Specific: what do you know about mother to infant/child transmission?

**Personal Questions**

1. Are you worried to get infected? Outside work? At work?

**Attitudes toward healthcare-related practices**

1. Have you ever treated hepatitis B positive patients in your practice before? If yes, how many?

2. Can you give a general overview how clinic/hospital usually treats hepatitis B patients?

Are there protocols, procedures, enough equipment? Any problems encountered in these situations?

3. In your experience, after you have treated someone with hepatitis B, what was their attitude like? Or how do you think they perceived their diagnosis, and their treatment and their follow up care?

**Reminders:**

- How did you feel while treating a patient with hepatitis B?

- Were you ever scared to touch a patient with hepatitis B? Why?

- Do you think that if a person tests positive, the doctor should inform the patient’s partner? Why?

- Do you think that hepatitis B patients should be made to pay for gloves, hepatitis kits, and other infection control supplies? Why?

- Do you think that patients with hepatitis B should be kept at a distance from other patients? Why?

- Do you think that clothes and linen used by hepatitis B patients should be disposed of or burned? Why?

- Do you think that people living with hepatitis B have a right to decide who should know about it?

**Attitudes towards People living with hepatitis B**

1. Do you believe that people with hepatitis B should still be able to marry? Why?

2. Is it ok for hepatitis B - positive women to get pregnant? Why?

3. Tell if you agree or disagree with these statements: (and why)

- Hepatitis B spreads due to immoral behavior.

- Women who get hepatitis B get what they deserve.

**Reminders:**

- What do you think are people more likely to get an hepatitis B infection?

- How would you feel if a coworker hepatitis B? Do you think he/she should tell their patient?

**Closing questions**

1. Are you vaccinated against hepatitis B?

2. Share experience, anything else to add?
